# Overdose prevention training with naloxone distribution in a prison in Oslo, Norway: a preliminary study

**DOI:** 10.1186/s12954-017-0200-z

**Published:** 2017-11-21

**Authors:** Aase Grønlien Petterson, Desiree Madah-Amiri

**Affiliations:** Norwegian Centre for Addiction Research, Institute of Clinical Medicine, University of Oslo, P.O. Box 1039 Blindern, 0315 Oslo, Norway

**Keywords:** Drug-dependent inmates, Opioid overdose, Naloxone training, Knowledge, Prison, Overdose prevention

## Abstract

**Background:**

Prison inmates face a ten times increased risk of experiencing a fatal drug overdose during their first 2 weeks upon release than their non-incarcerated counterparts. Naloxone, the antidote to an opioid overdose, has been shown to be feasible and effective when administered by bystanders. Given the particular risk that newly released inmates face, it is vital to assess their knowledge about opioid overdoses, as well as the impact of brief overdose prevention training conducted inside prisons.

**Methods:**

Prison inmates nearing release (within 6 months) in Oslo, Norway, voluntarily underwent a brief naloxone training. Using a questionnaire, inmates were assessed immediately prior to and following a naloxone training. Descriptive statistics were performed for main outcome variables, and the Wilcoxon signed-rank test was used to compare the participants’ two questionnaire scores from pre-and post-training.

**Results:**

Participating inmates (*n* = 31) were found to have a high baseline knowledge of risk factors, symptoms, and care regarding opioid overdoses. Nonetheless, a brief naloxone training session prior to release significantly improved knowledge scores in all areas assessed (*p* < 0.001). The training appears to be most beneficial in improving knowledge regarding the naloxone, including its use, effect, administration, and aftercare procedures.

**Conclusions:**

Given the high risk of overdosing that prison inmates face upon release, the need for prevention programs is critical. Naloxone training in the prison setting may be an effective means of improving opioid overdose response knowledge for this particularly vulnerable group. Naloxone training provided in the prison setting may improve the ability of inmates to recognize and manage opioid overdoses after their release; however, further studies on a larger scale are needed.

## Introduction

Approximately half of the European Union’s prison population has used illicit drugs at some point in their lives [1, 2]. The use of heroin is substantially greater among prisoners than the general population, with less than 1% of the general population and between 15 and 39% of prisoners using heroin [1]. Annual fatality rates in Norway are estimated to be around 70 per million, as compared to the European mean estimate of 17 deaths per million [3]. In Norway, approximately 260 people die of an overdose each year [4]. Nonfatal opioid overdoses are common [5], and the majority inject heroin [6]. In Norway, a study of 91,000 people post-prison release from 2000 to 2014 showed that a total of 0.5% (*n* = 493) died of an overdose within the first 6 months post-release and that 85% (*n* = 123) of the deaths during the first week were due to an overdose [7]. In the report *The Norwegian offender mental health and addiction*, the study suggests that half of the inmates used drugs for the last 6 months prior to incarceration [8]. Additionally, incarcerated people who use drugs have a three to eight-fold increase risk of drug-related death in the first weeks following release from prison [9]. The increased potential for overdosing upon release [9, 10] can be attributed to the enforced abstinence while incarcerated, coupled with reduced tolerance and relapse. Opioids combined with other legal and illegal drugs can nearly double this risk of death [11, 12]. Given this period of profound increased risk, targeted interventions are needed.

This elevated risk of drug-related death following liberation makes prisons a natural setting for opioid overdose prevention training with peer-administered naloxone. These trainings have been implemented upon prison release in the UK and the USA [13]. Following the implementation of Scotland’s national naloxone distribution program, they found a 36% reduction in overdose deaths during the first 4 weeks following prison inmates’ release [14] with an even greater reduction 5 years after the start of the program [15]. However, despite the apparent benefits of implementing overdose prevention training post-prison release, these programs remain relatively rare.

The majority of opioid overdoses occur within 1 to 3 h after injecting [16]. This window provides a chance to intervene if adequate training has been received beforehand [17–19]. Opioid overdose prevention trainings can significantly increase accurate recognition of an opioid overdose and equip those trained with the responses needed to prevent death [18, 20]. Proper training can also help battle incorrect responses, the product of hearsay and misinformation [21, 22]. This underlines the importance of providing overdose prevention training to those at risk of overdosing.

While prisoners are at risk of overdosing post-release, potential challenges for naloxone distribution specific to the prison setting need to be considered. Despite prisons being ideally positioned for overdose prevention initiatives, barriers to effective implementation exist [23, 24]. Some of these barriers include operational issues, identifying eligible inmates, and motivation for the willingness to attend the session, stigma among prisons and the prison staff, and an effective model to deliver training [23, 24]. Given these prison-specific barriers, prison-specific trainings need to be considered in attempt to optimize overdose prevention training and to reach this vulnerable group. Although a widespread take-home naloxone project began in Norway in 2014 [25], the project has been unable to be fully implemented within the prison settings. This is mainly due to many of the same barriers listed above. The aim of this study was to therefore assess the impact of a brief naloxone training on knowledge gained when conducted inside prison as preliminary investigation into scaling-up within the Norwegian prison setting.

## Methods

### Setting

Take-home naloxone is part of a government-supported intervention in Norway [26], wherein bystanders can receive naloxone from multiple distribution sites prescription free, and at no cost [25]. Although a safe injection facility has been in operation since 2005 in Oslo, drug use and trading remains illegal. In Norwegian prisons, half of the inmates are drug-dependent, and 1 in 4 reported injecting drugs daily before incarceration [8]. Further, 55% were under the influence of drugs when arrested for crimes related to drug sales, possession, smuggling, and driving under influence [8]. The study took place in a prison in Oslo, Norway, during a 2-month period in 2015. Oslo prison is publically funded and is one of the largest high security prisons in Norway. It can house up to 420 male individuals*.* Access to the inmates was granted following meetings with leadership and the researchers.

### Inclusion criteria

Inclusion criteria consisted of former or current opioid users, or people who were at risk of witnessing or experiencing an opioid overdose. To reach the people at highest risk of an opioid overdose, the study targeted those that were soon to be released (within the next 6 months). Further, participants must have been over 18 years of age, fluent in Norwegian or English, and had not received prior naloxone training.

### Participant characteristics

Participants were recruited via the health care staff based in the medical unit within the prison or by word-of-mouth. The medical unit inside prison provides medical care and offers either methadone or buprenorphine for opioid maintenance therapy (OMT). Inmates from different units were briefed on the study by the first author either during group meetings or by approaching them in their cell. Those that were interested were encouraged to participate. Participants were informed about the study, and those interested in participating provided signed consent.

The training took place in a one-on-one format in the medical unit. Several potential participants did not enroll in the study because of challenges related to resources, culture, access and time; however, details on those that refused were not collected. Participants were not reimbursed or incentivized for their time. A total of 31 inmates that fulfilled the study inclusion criteria volunteered to participate in the study.

### Instruments

This study utilized the Opioid Overdose Knowledge Scale (OOKS) developed for bystanders by Williams et al. [27]. The scale was originally designed for use alongside peer-administered intramuscular naloxone, and had a total score range of 0–45 points. However, in order to adapt it for intranasal naloxone use, items 5 and 6 were removed, resulting in the total score for this study of 0–39 points. Maximum possible scores for the scale’s four domains included risk (9), recognizing the signs of an overdose (10), actions to take when witnessing an overdose (11), and how to use naloxone (9). The questionnaire was translated into Norwegian for the purposes of this study by the first author. While translation of any kind risks altering the reliability and validity of a given research tool, the likelihood of the OOKS being compromised here is minimal because of the relative simplicity of the text translated. The questionnaire was not piloted within this setting prior to its use.

### Naloxone training

The training in the prison was led by the first author. Training curriculum used for the project is covered elsewhere [25, 28]. All training sessions took place in the prison’s medical unit office and took approximately 15–30 min to complete. Prior to the naloxone training, the participants were asked to complete the OOKS questionnaire. Next, they underwent a brief naloxone training session, where they learned to recognize and respond to an opioid overdose with naloxone, as well as how to assemble and use the device. During the training, misinformation and myths were identified and corrected where necessary. Immediately following their training, the participants were asked to complete the same questionnaire. The naloxone kit was placed with the participants’ personal items by prison staff to be received upon the day of their release. Follow-up regarding the participant’s use of naloxone was not completed in this study. All conversational techniques used were derived from motivational interviewing, both during the training and in the follow-up conversations [29, 30].

### Data analysis

Descriptive statistics were performed for the main outcome variables. The Wilcoxon signed-rank test was used to compare the participants’ two questionnaire scores from pre-and post-training. Based on Cohen’s criteria of effect size (r), this study interpreted 0.1 = small effect, 0.3 = medium effect, and 0.5 = large effect. Statistical analysis was performed using SPSS software version 22.

## Results

Participant characteristics are listed in Table 1. Nearly all participants had experience with overdoses, including having witnessed (*n* = 29, 93.5%) or experienced (*n* = 21, 67.7%) at least one overdose during their lifetime. Data on experiencing a recent overdose (e.g., within the last 12 months) was not collected. Half of the participants were receiving opioid maintenance treatment (OMT) prior to prison (*n* = 15, 48.3%), and many of the participants reported at least one risky behavior at some point in their lives, such as using drugs alone (*n* = 14, 45.2%), injecting (*n* = 14, 45.2%), or mixing opioids with other substances.

**Table 1:** Demographics and substance habits that study participants report prior to incarceration

	*N* (%)
Gender, males	31 (100)
Age, mean (SD)	35.6 (9.3)
Frequency of opioid use	
Daily	9 (29)
Almost daily	2 (6.5)
Previously	13 (41.9)
Never	7 (22.6)
Detoxification occurred in last 30 days	2 (6.5)
Receiving OMT	15 (48.3)
Uses drugs alone	
Sometimes	7 (22.6)
Often	4 (12.9)
Most of the time	2 (6.5)
All of the time	1 (3.2)
Never	2 (6.5)
Missing/not applicable	15 (48.3)
Mixes opioids with^a^	
Alcohol	5 (16.1)
Benzodiazepines	10 (32.3)
Cocaine	2 (6.5)
Methamphetamine	13 (41.9)
GHB/GBL	4 (12.9)
Other	2 (6.5)
Mode of administration	
Injecting	14 (45.2)
Smoking	1 (3.2)
Snorting	1 (3.2)
No response	15 (48.4)
Witnessed an overdose previously	
1–10 times	13 (41.9)
11–20 times	5 (16.1)
More than 20 times	11 (35.5)
Never	2 (6.5)
Experienced an overdose personally	
1–10 times	14 (45.2)
11–20 times	4 (12.9)
More than 20 times	3 (9.6)
Never	10 (32.3)

*SD* standard deviation, *OMT* opioid maintenance treatment, *CPR* cardio-pulmonary resuscitation, *GHB/GBL* gamma hydroxybutyrate/gamma butyrolactone

^a^Multiple responses possible

### Opioid Overdose Knowledge Scale

The participants had a high baseline knowledge of risk factors (mean = 8.1), signs of an overdose (mean = 8.5), and actions to take when responding to an overdose (mean = 10.1) (Table 2). Nonetheless, the educational session elicited significant improvements in all domains and with a large effect size (*r* = 0.88). The most substantial improvements were seen in the knowledge on the use of naloxone (*r* = 0.85) and risk factors (*r* = 0.74).

**Table 2:** Self-reported change in knowledge pre- and post-training

Knowledge domains (points)	Pre-training mean (SD)	Post-training mean (SD)	Mean difference	Wilcoxon Z	*P* value
Risk factors (0–9)	8.1 (1.1)	9 (9)	0.94	− 4.128	< 0.001
Signs (0–10)	8.5 (1.2)	9.8 (0.7)	1.36	− 4.005	< 0.001
Actions (0–11)	10.1 (1.2)	10.9 (0.3)	0.8	− 3.372	< 0.001
Naloxone (0–9)	5.5 (1.9)	9 (0.2)	3.52	− 4.730	< 0.001
Total (0–39)	32.1 (3.4)	38.7 (0.7)	6.58	− 4.873	< 0.001

*SD* standard deviation

### Risk

All participants were found to have a high baseline knowledge of the risk factors that increase the probability of an overdose, although more than half (*n* = 18, 58%) of the participants did not know that a long history of heroin use contributed to increased risk.

### Signs

The participants had a high baseline knowledge of indicators of an opioid overdose and could identify symptoms that do not occur during an overdose. These included bloodshot eyes, agitated behavior, and rapid heartbeat. However, almost a third of participants (*n* = 9, 29%) incorrectly believed fitting (small seizures) to be a sign of an opioid overdose. Of the group, 39% (*n* = 12) did not recognize very small pupils as an indicator of the “opioid overdose triad” (along with reduced consciousness and respiratory depression), and only 35% (*n* = 11) recognized deep snoring as a sign of an opioid overdose.

### Action

Most of the participants had carried out important actions toward managing an opioid overdose. All participants responded that they would call an ambulance, check for breathing, and stay with the person until the ambulance arrived. Ninety-seven percent (*n* = 30) would put the victim in the recovery position and make sure the airways were cleared and most (*n* = 28, 90%) would give pulmonary resuscitation. Almost no one (*n* = 30, 97%) would inject the victim with milk or salt solution, and only one (3%) would put the person in bed to “sleep off” the overdose. Nearly all of the participants (*n* = 30, 97%) would give naloxone if it were available. However, one quarter of participants (*n* = 8, 26%) would give stimulants to an overdose victim in the form of cocaine or coffee, and one third (*n* = 11, 35%) would put the person in a bath of cold water.

### Naloxone use

Nearly all of the participants (*n* = 30, 97%) knew about the uses of naloxone. However, 61% (*n* = 19) of them were not aware of how long it takes for naloxone to take effect, and 84% (*n* = 26) did not know for how long these effects persist. Of the group, 23% (*n* = 7) did not know that if the first dose had no effect, a second dose could be given. Sixty-eight percent (*n* = 21) did not know that a person can overdose again after having received naloxone, and 61% (*n* = 19) were not aware that naloxone has a shorter duration than many opioids. A third of participants (*n* = 11, 35%) did not know that naloxone could provoke withdrawal symptoms. Reassuringly, 90% (*n* = 28) would call an ambulance even though they knew how to manage an overdose and the same number understood that it was not wise for the overdose victim to drink alcohol or take sleeping tablets afterwards.

## Discussion

The aim of this study was to assess knowledge of opioid overdoses among inmates at risk of witnessing or experiencing an overdose before and after a brief training session about naloxone. Half of the participants were receiving OMT prior to prison, indicating their relevance as a target group to receive opioid overdose prevention training. The naloxone training session significantly improved inmates’ knowledge in all four domains assessed. The areas with the greatest post-training improvement were for the use and effect of naloxone, how to administer it, and aftercare procedures.

The participant characteristics support that this study reached a high-risk group (incarcerated individuals who have experienced or witnessed an overdose). Nearly every participant reported that they previously had witnessed an overdose and almost half had experienced between 1 and 10 personally. Participants reported responses that reflected misconceptions about overdoses. This was similar to Wakeman et al. who found that these methods included shaking/slapping the victim (20%), putting the person in a bath of cold water (41%), and injecting the person with salt water (16%) [31]. Additionally, they did not use naloxone because they did not have it available, but nearly 90% reported willingness to learn about overdose prevention and naloxone administration [31]. More than half of the participants in this study answered that a long history of drug use could decrease the tolerance of drugs rather than increase it. Many of the participants also failed to identify pinpoint pupils and deep snoring as common signs of an opioid overdose. While overall, the participants’ baseline knowledge was high, similar to what others have found [27]; the misconceptions suggest the necessity of overdose prevention training in prisons.

The findings from this study are consistent with the existing literature [13], much of which highlights the effectiveness of education in improving knowledge of how to manage an opioid overdose and how to distinguish its symptoms from those of other medical conditions [18–20, 32–34]. The majority of witnesses to an opioid overdose are willing to take countermeasure actions [35], even though some of these actions might be misinformed [22, 36]. The findings are in accordance with previous research suggesting that peer-administered naloxone, following a brief educational intervention, may have a positive impact in providing skills to a vulnerable group upon their release from prison [14, 18].

This study experienced limitations. First, within the participating prison, difficulties with identifying eligible inmates may have affected those that participated. Some of the participants had competing priorities, including school, prison-duties, and appointments that interfered with their time available to participate. There was also limited awareness among prison staff regarding naloxone. This could have occurred due to confusion about the nature of the study among prison staff, or because of stigma among the staff, some of whom expressed concerns that naloxone might encourage drug-using behavior once the inmates were released. This limited awareness among prison officers regarding naloxone may have affected the inmate’s desire to participate. Participants expressed concerns with the training and that it could mark them as “not rehabilitating.” These challenges are consistent with the existing literature [23, 24]. It is possible that this perceived stigma and hesitance could have introduced selection bias, although the use of an independent researcher (not prison staff) may have minimized this bias. Second, participants may have experienced a social desirability bias, wherein participants reported answers that they believe to be the most acceptable [37], but again this may have been minimized due to the use of independent staff. Even so, the changes in the scores pre- and post-training give an indication of knowledge gained and are not likely influenced by the study’s limitations. The results from this study are likely generalizable to the other similar prison and societal settings, particularly in Norway.

## Conclusions

This study demonstrates that naloxone training in the prison setting may be an effective means of improving knowledge about opioid overdoses to a vulnerable group. A brief naloxone training session was found to improve knowledge scores across all domains (risk, signs, action, naloxone use), thereby substantiating the utility and appropriateness of opioid overdose prevention training prior to the inmates’ release from prison. The naloxone program in Norway has previously been unable to be implemented on a large-scale within the prison settings. As the program expands, emphasis on establishing within prisons is a priority. The findings from this study provide preliminary information regarding the transfer of knowledge to inmates; however, further studies will be needed in other settings to assess the impact of such interventions on opioid overdoses for those recently released from prison and the feasibility of establishing within the prison setting.

## Abbreviations

CRP: Cardio-pulmonary resuscitation
GHB/GBL: Gamma hydroxybutyrate/gamma butyrolactone
OMT: Opioid Maintenance Treatment
OOKS: Opioid Overdose Knowledge Scale
SD: Standard deviation
SPSS: Statistical Package for the Social Sciences
